# Ceftriaxone‐Associated Cholelithiasis in a Premature Toddler From Ethiopia: A Case Report

**DOI:** 10.1155/carm/6644319

**Published:** 2026-04-24

**Authors:** Kaleb Assefa Berhane, Selamawit Tesfaye, Meron Zeleke, Adu Dufera Moti, Dawit Taye Endalew, Lela Alemayehu Gebeyehu, Amanuel Getu Gebreselassie

**Affiliations:** ^1^ Department of Internal Medicine, Adera Medical and Surgical Center, Addis Ababa, Ethiopia; ^2^ School of Medicine, St. Paul’s Hospital Millennium Medical College, Addis Ababa, Ethiopia, sphmmc.edu.et

**Keywords:** case report, ceftriaxone, cholelithiasis, gallstones, pediatric, pseudolithiasis

## Abstract

Ceftriaxone‐associated cholelithiasis is an uncommon but well‐recognized adverse effect in pediatric patients, resulting from precipitation of ceftriaxone–calcium complexes within bile. Although often asymptomatic and transient, its radiologic appearance may mimic true gallstones and lead to unnecessary surgical intervention. We report a 2‐year‐old male child, born prematurely at 34 weeks of gestation, who was incidentally diagnosed with gallbladder stones during follow‐up evaluation after multiple hospital admissions for dehydration and presumed infections treated with high‐dose intravenous ceftriaxone. The child was asymptomatic for biliary disease, with normal physical examination and laboratory findings. Abdominal ultrasonography demonstrated multiple mobile echogenic foci with posterior acoustic shadowing, the largest measuring 1.1 cm, without evidence of inflammation or biliary obstruction. Given the absence of symptoms and identifiable hemolytic or metabolic disorders, conservative management with close follow‐up was adopted. Repeat ultrasonography after 12 weeks showed complete spontaneous resolution of the gallstones. This case highlights the importance of recognizing ceftriaxone‐associated cholelithiasis in young children to help avoid unnecessary surgical intervention in appropriately selected asymptomatic patients through appropriate conservative management.

## 1. Introduction

Cholelithiasis refers to the presence of gallstones within the gallbladder or, less commonly, within the intrahepatic or extrahepatic bile ducts. While gallstones are frequently encountered in adults, pediatric cholelithiasis remains relatively rare, with reported prevalence ranging from 0.13% to 1.9% [1, 2]. The earliest reported case of pediatric cholelithiasis was described by Gibson in 1737. Children, however, are more likely than adults to present with symptoms, and the increasing use of ultrasonography has led to a rise in incidental detection of asymptomatic cases [3, 4].

The etiologic spectrum of pediatric cholelithiasis differs substantially from that of adults. Hemolytic disorders remain the most common cause, accounting for approximately 20%–30% of cases. In recent years, nonhemolytic causes have gained prominence, including prematurity, total parenteral nutrition, ileal disease, congenital hepatobiliary anomalies, obesity, medication exposure, and idiopathic factors. Among medications, ceftriaxone, a widely used third‐generation cephalosporin, has been increasingly recognized as a reversible cause of gallbladder stone or sludge formation in children [3–5].

Despite numerous reports of ceftriaxone‐associated biliary cholelithiasis, diagnostic and management challenges persist, particularly in resource‐limited settings where differentiation between transient drug‐induced stones and true gallstones may be difficult. This case is reported to emphasize the spontaneous resolution of ultrasonographically evident gallstones in a young, premature child following conservative management, reinforcing the importance of avoiding unnecessary surgical intervention.

## 2. Case Presentation

A 2‐year‐old male child, born prematurely at 34 weeks of gestation to a nulliparous mother with preeclampsia, required admission to the neonatal intensive care unit (NICU) for supportive care related to prematurity. His neonatal course was otherwise uneventful, and he was discharged in stable condition with normal developmental progress thereafter.

Beginning at 15 months of age, the child experienced multiple recurrent episodes of diarrhea and nonbilious vomiting, frequently diagnosed as acute gastroenteritis with associated severe dehydration. On several of these occasions, he also presented with respiratory symptoms suggestive of lower respiratory tract infections. During these episodes, he was managed with repeated courses of ceftriaxone (100 mg/kg/day once daily) and intravenous fluids and received supportive care. There was no history of exposure to other hepatobiliary medications or use of total parenteral nutrition.

Two weeks following the most recent illness episode, the child was brought for follow‐up evaluation to our outpatient clinic with an abdominal ultrasound report revealing an incidental finding of gallstones. The mother reported that the child had no yellowish discoloration of the skin, pale stools, dark urine, right upper quadrant pain, or postprandial discomfort and irritability. His past surgical history was unremarkable.

On examination, he appeared well and was growing appropriately for age. He was hemodynamically stable, and physical examination was unremarkable, including a soft, nontender abdomen without organomegaly.

**FIGURE 1:**
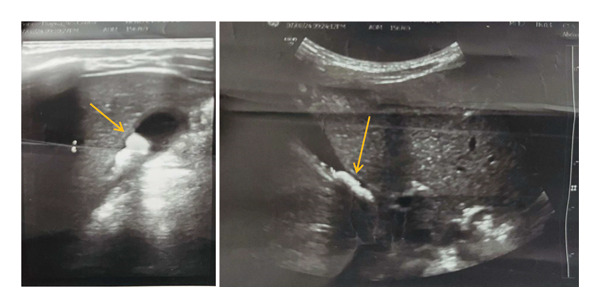
Sagittal and subcostal ultrasound views showing multiple gallstones within the gallbladder.

Laboratory investigations, including complete blood count, liver function tests, serum amylase, lipase, and renal function tests, were all within normal limits. Abdominal ultrasound showed multiple intraluminal echogenic foci with posterior acoustic shadowing, the largest measuring 1.1 cm (Figure 1) without gallbladder wall thickening, pericholecystic fluid, or biliary duct dilation. No structural hepatobiliary anomalies were identified.

Further hematologic and metabolic evaluations were unremarkable. A peripheral blood smear showed normocytic, normochromic red cells with no abnormal morphology. Reticulocyte count, LDH, and haptoglobin levels were within normal range. Hemoglobin electrophoresis did not reveal features of hemoglobinopathies. Serum ferritin, lipid profile, fasting glucose, and vitamin B12 were normal.

Given the absence of symptoms and the likely association with prior ceftriaxone exposure, a conservative approach was taken. The child was monitored clinically without intervention. Follow‐up imaging performed 12 weeks later showed complete resolution of the gallstones, consistent with transient, ceftriaxone‐associated cholelithiasis.

## 3. Discussion

This case illustrates asymptomatic, transient cholelithiasis in a young child with a history of prematurity and repeated exposure to high‐dose ceftriaxone, with complete spontaneous resolution following conservative management.

Ceftriaxone‐associated biliary cholelithiasis, first reported in 1986, is a well‐documented adverse drug reaction with a reported incidence of < 0.1% in the general population. Ceftriaxone is a third‐generation cephalosporin widely used in pediatric practice because of its broad‐spectrum activity, prolonged plasma half‐life, and favorable safety profile. Approximately 60% of the drug is excreted renally and 40% via the biliary system [5–7]. Due to this substantial biliary excretion, ceftriaxone can concentrate in bile at levels 20–150 times higher than serum concentrations, where it may bind calcium to form insoluble complexes, resulting in biliary pseudolithiasis [3]. It presents as echogenic material—sludge or pseudoliths—within the gallbladder, which closely mimics true gallstones on ultrasonography. These transient sonographic findings are termed “ceftriaxone pseudolithiasis” due to their morphologic similarity to gallstones and their typically rapid resolution following discontinuation of the antibiotic [6]. Although ceftriaxone‐associated biliary precipitates are often termed “pseudolithiasis” due to their transient nature, it is important to recognize that spontaneous resolution alone does not definitively distinguish these from true gallstones. Small gallstones may pass through the biliary system into the duodenum, resulting in similar radiologic resolution.

Several studies have highlighted ceftriaxone as a significant risk factor for pediatric cholelithiasis. In a study conducted in Iran, ceftriaxone was identified as the most common predisposing factor, accounting for 27.3% of cases. Similarly, a multicenter study in Italy reported ceftriaxone‐related cholelithiasis in 6% of pediatric patients [2]. A study from Egypt involving 35 children with gallstones found that 5 cases (14.3%) were attributed to ceftriaxone therapy, of which 4 were managed conservatively. The same study reported 2 cases (5.7%) associated with prematurity, both asymptomatic and also managed conservatively [8]. Consistent findings were reported in an Indian study of 56 pediatric patients, where prematurity was identified as a notable risk factor in 14 cases (25%) [9]. In our case, prematurity and repeated high‐dose ceftriaxone administration in the context of dehydration likely contributed synergistically to stone formation.

Management of cholelithiasis in children depends on symptom severity, underlying etiology, gallbladder anatomy, presence of biliary inflammation, associated anatomical abnormalities, and patient age. Asymptomatic gallstones smaller than 10 mm that are freely mobile within the gallbladder generally warrant conservative management after evaluation for underlying conditions such as hemolytic disorders. These patients can be safely monitored with periodic ultrasonography unless complications develop [3].

Most cases of ceftriaxone‐associated cholelithiasis are asymptomatic and incidentally detected on imaging, as in our patient. A review of English‐language literature indexed in PubMed from 1991 to 2022 identified only 30 reported cases of symptomatic ceftriaxone‐associated cholelithiasis, 16 of which occurred in pediatric patients [4, 6]. Cholecystectomy is typically reserved for children with persistent or symptomatic gallstones, particularly when spontaneous resolution is unlikely or complications occur [2].

In Ethiopia the first reported pediatric cholelithiasis involved a 9‐month‐old infant with symptomatic gallstones and no clear risk factors, requiring cholecystectomy [10]. In contrast, our case describes a 2‐year‐old male with asymptomatic cholelithiasis associated with ceftriaxone exposure and prematurity, which resolved spontaneously with conservative management.

The onset of ceftriaxone‐associated cholelithiasis is typically reported 10–20 days after initiation of therapy, although detection as early as one week has been described. In asymptomatic cases identified incidentally, discontinuation of ceftriaxone is generally unnecessary, whereas cessation is recommended when symptoms develop. Resolution usually occurs within 2 weeks of stopping the drug, although persistence for up to 90 days and rarely as long as 7 months has been reported. Notably, patients whose ceftriaxone‐associated cholelithiasis resolves tend to be significantly younger than those with gallstones of other etiologies [4, 7]. In our case, gallbladder stones resolved spontaneously within 3 months, without the need for surgical intervention.

## 4. Conclusion

This case underscores ceftriaxone‐associated cholelithiasis as a reversible and self‐limiting condition in young children, particularly in the presence of risk factors such as prematurity and repeated high‐dose exposure. Recognition of this entity is essential, as its ultrasonographic appearance may mimic true gallstones. Careful clinical correlation and conservative management with follow‐up imaging can allow spontaneous resolution and help avoid unnecessary surgical intervention.

**Nomenclature**
LDH: Lactate dehydrogenase

## Author Contributions

Kaleb Assefa Berhane: conceptualization, data curation, investigation, project administration, writing–original draft, and writing–review and editing.

Selamawit Tesfaye: conceptualization, data curation, investigation, resources, and writing–review and editing.

Meron Zeleke: conceptualization, data curation, writing–original draft, and writing–review and editing.

Adu Dufera Moti: conceptualization, methodology, visualization, investigation, and writing–review and editing.

Dawit Taye Endalew: conceptualization, methodology, visualization, investigation, and writing–review and editing.

Lela Alemayehu Gebeyehu: conceptualization, methodology, visualization, investigation, and writing–review and editing.

Amanuel Getu Gebreselassie: conceptualization, methodology, visualization, investigation, and writing–review and editing.

## Funding

No funding was received for this research.

## Disclosure

All authors reviewed and approved the final version of the manuscript.

## Ethics Statement

The study is exempt from ethical approval in our institution. All the information obtained was held confidential and used only for the intended purpose.

## Consent

Written informed consent was obtained from the patient for publication of this case report and the accompanying images.

## Conflicts of Interest

The authors declare no conflicts of interest.

## Data Availability Statement

The data that support the findings of this study are available from the corresponding author upon reasonable request.
